# Cloning of a CHS gene of *Poncirus trifoliata* and its expression in response to soil water deficit and arbuscular mycorrhizal fungi

**DOI:** 10.3389/fpls.2022.1101212

**Published:** 2022-12-20

**Authors:** Zhen Liu, Shen Cheng, Xiao-Qing Liu, Kamil Kuča, Abeer Hashem, Al-Bandari Fahad Al-Arjani, Khalid F. Almutairi, Elsayed Fathi Abd_Allah, Qiang-Sheng Wu, Ying-Ning Zou

**Affiliations:** ^1^ College of Horticulture and Gardening, Yangtze University, Jingzhou, Hubei, China; ^2^ Department of Chemistry, Faculty of Science, University of Hradec Kralove, Hradec Kralove, Czechia; ^3^ Botany and Microbiology Department, College of Science, King Saud University, Riyadh, Saudi Arabia; ^4^ Plant Production Department, College of Food and Agricultural Sciences, King Saud University, Riyadh, Saudi Arabia

**Keywords:** arbuscular mycorrhiza, chalcone synthase, drought, trifoliate orange, flavonoid

## Abstract

Flavonoids are secondary metabolites widely found in plants with antioxidants, of which chalcone synthase (CHS) is a key enzyme required in flavonoid synthesis pathways. The objective of this study was to clone a *CHS* gene from trifoliate orange (*Poncirus trifoliata*) and analyze its biological information and partial functions. A PtCHS gene (NCBI accession: MZ350874) was cloned from the genome-wide of trifoliate orange, which has 1156 bp in length, encoding 391 amino acids, with a predicted protein relative molecular mass of 42640.19, a theoretical isoelectric point of 6.28, and a lipid coefficient of 89.82. The protein is stable, hydrophilic, and high sequence conservation (92.49% sequence homology with CHS gene of other species). *PtCHS* was highly expressed in stems, leaves and flowers, but very low expression in roots and seeds. Soil water deficit could up-regulate expressions of *PtCHS* in leaves. An arbuscular mycorrhizal fungus, *Funneliformis mosseae*, significantly increased plant biomass production, CHS activity, expressions of *PtCHS*, and total flavonoid content in leaves and roots, independent of soil water status. Total flavonoids were significantly positively correlated with *PtCHS* expression in leaves only and also positively with root mycorrhizal colonization. Such results provide insight into the important functions of *PtCHS* in trifoliate orange.

## Introduction

Flavonoids are important secondary metabolites of horticultural plants, which have antioxidant, antibacterial, and anti-inflammatory properties (Shen et al., 2022). In addition, flavonoids have some applications in the food industry, cosmetics, and pharmaceutical industries (Dias et al., 2021). The synthesis of flavonoids is accomplished with the joint participation of various enzymes, among which chalone synthase (CHS) is the first key enzyme in the flavonoid biosynthesis pathway (Yonekura et al., 2019). CHS is to catalyze the synthesis of naringenin chalcone from p-coumaroyl CoA and malonyl CoA, which is further derived and transformed into various flavonoid compounds under the catalysis of chalcone isomerase (CHI) (Han et al., 2016, Yahyaa et al., 2017). CHS is widely found in higher plants, and the expression of its gene family members is tissue-specific and time-specific (Chen et al., 2017; Sepiol et al., 2017). Environmental stress such as temperature stress and drought stress induces CHS expressions to promote the accumulation of flavonoids (Dao et al., 2011; Li et al., 2020; Yin et al., 2020). Hence, the gene plays an important role in plant response to stress and regulation of flavonoid synthesis (Dixon and Paiva, 1995; Gläßgen et al., 1998). CHS gene has been cloned in many crops, such as rice (Han et al., 2017), mulberry (Wang et al., 2017), and citrus (Wang et al., 2018), but there is no report in trifoliate orange (*Poncirus trifoliata*). Hu et al. (2019) reported that overexpression of a *CHS* gene from tobacco (*Nicotiana tabacum*) could mitigate drought-induced oxidative damage and thus enhanced drought tolerance. Nakabayashi et al. (2014) also observed in *Arabidopsis thaliana* that excessive accumulation of flavonoids was the key to enhance drought tolerance of plants. Therefore, up-regulated expression of flavonoid biosynthetic genes and accumulation of flavonoids are important mechanisms for drought tolerance in plants (Ma et al., 2014).

Trifoliate orange, belonging to the genus *Poncirus* in the Rutaceae family, has the advantages of resistance to root rot, tristeza virus, cold, and drought tolerance, and is the most widely used rootstock in citrus production (Wu et al., 2010). In addition, trifoliate orange is a citrus relative with abundant bioactive substances, such as flavonoids, carotenoids, and terpenoids (Gao et al., 2018; Sharma et al., 2019). All citrus plants can produce flavonoids (Ghasemi et al., 2009), such as sweet orange, pomelo, and lemon. At present, more than 60 flavonoids have been identified in citrus (Tripoli et al., 2007). Flavonoids of citrus not only have antioxidant, anti-inflammatory, anti-tumor, and other functions (Song et al., 2017; Mahato et al., 2018), but also play an important role in the coloring of flowers, fruits and leaves, abiotic and biotic tolerance, auxin transport, nutritional value, and fruit flavor (Ferreyra et al., 2012; Flamini et al., 2013; Gabriele et al., 2017).

Soil water deficit (SWD) is one of abiotic stress restricting crop growth, which can lead to reduced crop growth and yield, and even crop death in severe cases (Kunert et al., 2016). Arbuscular mycorrhizal fungi (AMF) in soil form a reciprocal symbiosis with plant roots (He et al., 2019), which can absorb water and nutrients from the soil to host plants for their growth and enhance SWD tolerance, along with high utilization value in agricultural production (Wu et al., 2013; Zou et al., 2017). Studies have shown that appropriate SWD promoted flavonoid accumulation in plants (Ma et al., 2014), and AMF promoted photosynthesis, nutrient absorption, and various secondary metabolite levels in plants under SWD (Cheng et al., 2022). We hypothesized that AMF up-regulates the expression level of *CHS* in trifoliate orange under drought and thus promotes the level of flavonoids, which is beneficial for mycorrhizal plants to tolerate SWD.

In order to confirm the above hypothesis, we cloned a CHS gene from *P. trifoliata*, analyzed the physicochemical properties of the protein, constructed an evolutionary tree, and analyzed the relative expression of *PtCHS* gene in leaves and roots under SWD and AMF inoculation.

## Materials and methods

### Cloning of PtCHS gene

Total RNA was extracted from leaves, stems, roots, flowers, fruits, and seeds of trifoliate orange grown in a citrus orchard of Yangtze University using the TaKaRa MiniBEST Universal RNA Extraction Kit. After checking the concentration and purity of the extracted RNA, the PrimeScript™ RT Reagent Kit with gDNA eraser was used to reverse-transcribe RNA into cDNA using a Bio Photometer Plus PCR (6132, Eppendorf, Germany).

The Arabidopsis CHS gene (NCBI accession number: AT5G13930) was used as the reference sequence, and the BLASTP of the trifoliate orange genome database was used to search CHS gene. A pair of primers (F: 5’-CCAAGCACGAGCCTCAAAAC-3’; R: 5’-ACAGCACACCCCAATCTAGC-3’) was designed using Primer premier 5.0 software to amplify the full-length sequence of the gene, in which planta max super-fidelity DNA polymerase kit (Vazyme Biotech Co., Ltd, Nangjing, China) was used under the condition of 95°C for 3 min, 95°C for 15 s, 56°C for 15 s, and 72°C for 3 min with 35 cycles.

After the PCR reaction, the product fragments were recovered and the target fragments were ligated and transformed using the pEASY®-Blunt Zero Cloning Kit (Beijing TransGene Biotech Co., Ltd, Beijing, China). Positive clones were screened on LB plates coated with ampicillin, and then sequenced by Department of Qingke Biotechnology Co., LTD. (Wuhan, China). The sequencing results were spliced by DNAMAN 6.0, and the full-length sequences of the cloned genes were obtained by analysis and comparison using BLAS of NCBI.

### Bioinformatics analysis of PtCHS

Multiple alignments of amino acid sequences were performed using the DNAMAN (V6.0). The amino acid sequence of PtCHS gene was constructed by Mega-X, and the neighbor-joining (NJ) method was used to generate the evolutionary tree. Bootstrap was used to validate the phylogenetic tree, and the number of replicates was defined as 1000. According to the online tool (https://swissmodel.expasy.org/) the gene structure was predicted. SOPMA (http://npsa-pbil.ibcp.fr/cgi-bin/npsa_automat.pl?page=npsa_sopma.html) and Swiss-Model (http://swissmodel.expasy.org/interactive) were used for secondary and tertiary structure prediction of the protein, respectively. The protein subcellular localization was predicted using the online WoLF PSORT (https://wolfpsort.hgc.jp/) to predict.

### Plant culture and AMF inoculation

Four-leaf-old trifoliate orange seedlings grown in sterilized river sand were transplanted into a plastic pot containing 2.5 kg of autoclaved soil and sand mixture (3 : 1, v/v). At the same time, the 120 g inoculums of *Funneliformis mosseae* (BGC XZ02A) (22 spore/g) were applied near the rhizosphere. Uninoculated (-AMF) plants were treated with 120 g of autoclaved inoculums and a filtrate (25 µm filter) of 2 mL of mycorrhizal inoculums to maintain similar microbial community composition except for the *F. mosseae*. After transplantation, all treated plants maintained the soil moisture at well-watered (WW) (75% of the maximum field water capacity) for their growth. After 8 weeks, half of the inoculated and uninoculated plants were subjected to SWD (55% of the maximum field water capacity) for 10 weeks, and the other half continued to grow in the soil with WW status for another 10 weeks. Soil moisture was monitored daily by weighing method, and the loss of soil water was replenished in time. The plants were grown in a greenhouse, where the environmental conditions have been described by Zhang et al. (2020). Therefore, this experiment consisted of four treatments: the seedlings inoculated with AMF and grown in WW (WW+AMF); the seedlings inoculated without AMF and grown in WW (WW-AMF); the seedlings inoculated with AMF and grown in SWD (SWD+AMF); the seedlings inoculated without AMF and grown in SWD (SWD-AMF). Each treatment was repeated six times.

### Determination of plant biomass production and root mycorrhizal colonization

At plant harvest, the biomass of the whole plant was weighted, frozen with liquid nitrogen, and immediately stored at -80°C for RNA extraction. Root mycorrhizae were stained according to the method described by Phillips and Hayman (1970). About 1-cm-long root segments were incubated in 10% KOH solution at 95°C for 100 min, rinsed with distilled water, bleached with 10% hydrogen peroxide solution for 15 min, acidified with 0.2 mol/L hydrochloric acid for 10 min, and stained with 0.05% trypan blue in lactate phenol for 1 min. The mycorrhizal colonization was observed under a microscope and calculated as the percentage of length of AMF-colonized root segments versus total length of observed root segments.

### Determination of CHS activity

The 0.5 g of fresh leaf and root samples were ground in 5 mL of 0.1 mol/L phosphate buffer (pH 7.6) in an ice bath and then centrifuged at 10,000×*g* for 10 min at 4°C. The supernatant was used as the crude extract for CHS activity determination. CHS activity was determined by the Enzyme-Linked Immunosorbent Assay (ELISA) according to the user manual, where the plant CHS kit (ml036296) was provided by Shanghai Enzyme Linked Biotechnology Co., Ltd (Shanghai, China).

### Relative expressions of PtCHS

Total RNA extraction from leaves and roots was performed according to the above procedure. The primer sequence of the gene was designed in Primer Premier 5.0 Software according to the full-length gene sequences obtained by sequencing, where the sequences were 5’-GTCTAAACTCGGCCTCAAAGA-3’ (forward primer) and 5’-TCTCGTCAAGGATGAACAGAAC-3’ (reversed primer). After reverse transcription of RNA into cDNA, the β-actin was used as the reference gene for qRT-PCR, based on the 2 × AceQ qPCR SYBR Green Master Mix (Aidlab, Beijing, China). There were three biological replicates for each treatment. The 2^-ΔΔCt^ method was used to calculate the relative expression of *PtCHS*, with the WW-AMF treatment as the control.

### Determination of total flavonoid content of leaves and roots

Plant total flavonoid content was assayed with the protocol of Liu et al. (2022). The 0.2 powdered samples of leaves and roots were extracted with 8 mL of 70% ethanol under ultrasonic conditions for 60 min, and centrifuged at 10,000×g for 10 min at 4°C. The extraction was repeated twice, and the supernatant was concentrated, evaporated to remove the ethanol, and added with methanol. A total of 10 mL reaction solution consisted of distilled water, 5% NaNO_2_, 10% AlCl_3_, 1 mol/L NaOH, and 0.5 mL of the tested solution, and their absorbance was measured at 510 nm, where the rutin was used as the standard.

### Data analysis

The data obtained were statistically analyzed under SAS software, where ANOVA as well as Duncan’ new multiple range test at 0.05 levels were performed for significance between treatments.

## Results

### Physicochemical properties of PtCHS protein

**Figure 1:**
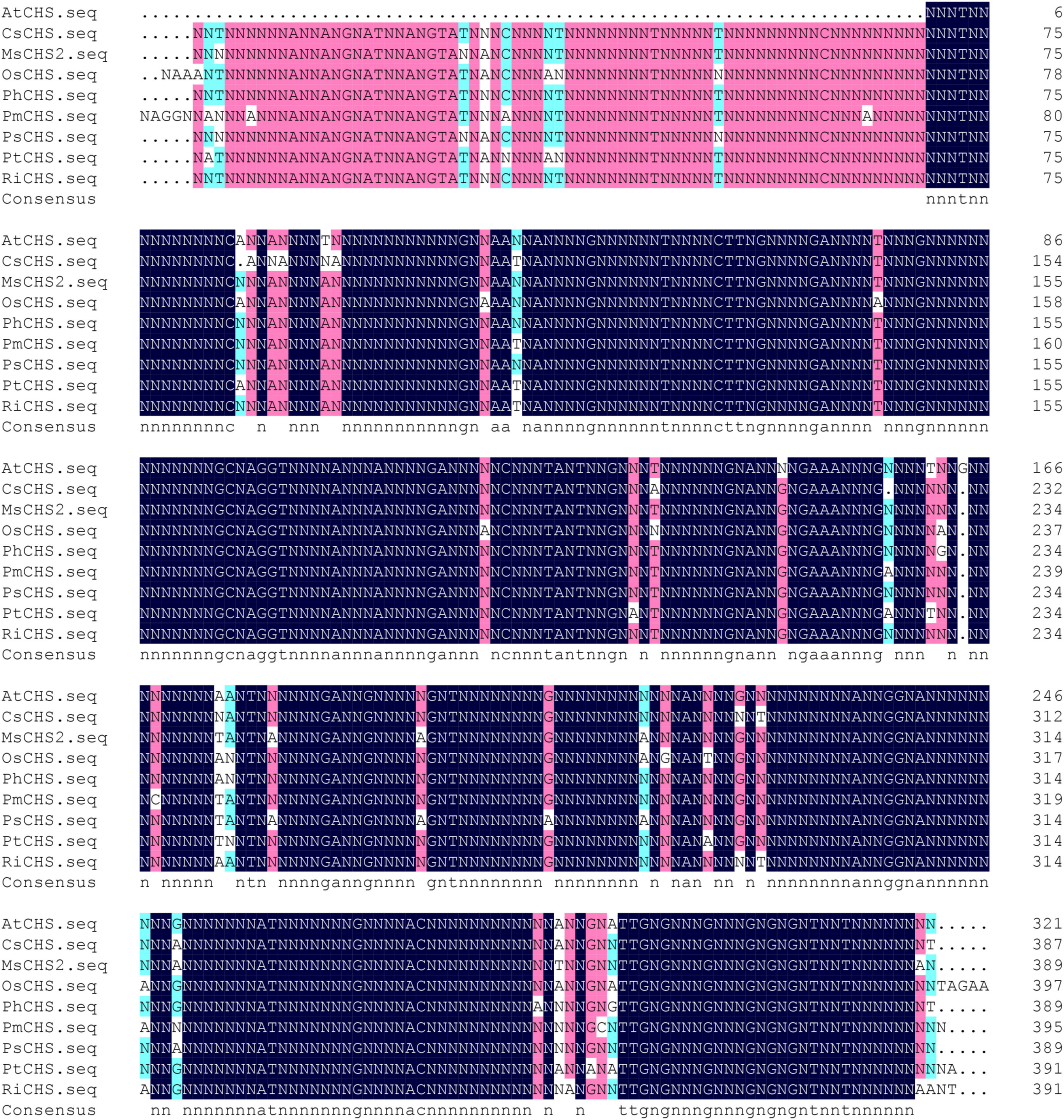
Sequence of PtCHS and its alignment with CHS gene of other plants. AtCHS, *Arabidopsis thaliana*; CsCHS, *Camellia sinensis*; MsCHS2, *Medicago sativa*; OsCHS, *Oryza sativ*; PhCHS, *Petunia* × *hybrida*; PmCHS, *Picea mariana*; PsCHS, *Pisum sativum*; PtCHS, *Poncirus trifoliata*; RiCHS, *Rubus idaeus*.

Sequencing results showed that the protein had 1156 bp in length (**Figure 1**), contained a complete open reading frame, and encoded 391 amino acids, along with NCBI accession number MZ350874.

**Figure 2:**
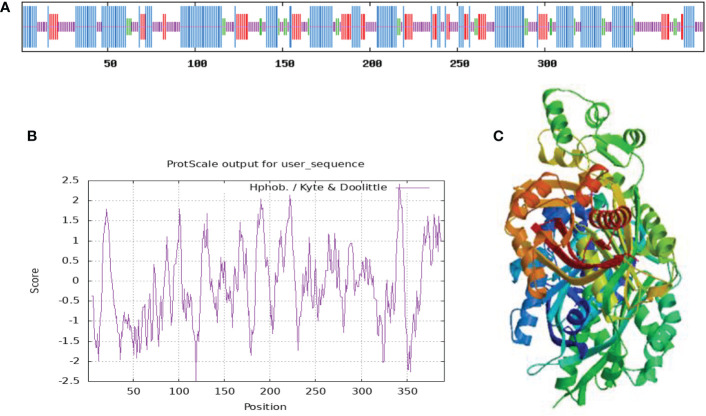
Hydrophilic and hydrophobic properties **(A)**, secondary structure **(B)**, and tertiary structure **(C)** of PtCHS protein.

The information of the protein predicted by ProtParam online tool (https://www.expasy.org/resources/protscale) showed that the relative molecular mass of this protein is 42640.19, the theoretical isoelectric point is 6.28, the molecular formula is C_1898_H_3037_N_507_O_567_S_19_, and the protein is not stability index of 35.00, indicating that the protein is stable. In addition, the protein had a relatively high aliphatic index of 89.82, which allows the protein to have good stability in different environments and facilitates its normal function, and a grand average hydrophilicity of -0.105. PortScale online tools (https://www.expasy.org/resources/protscale) in the prediction of protein hydrophilic/hydrophobic properties showed that amino acid sequence in the 119^th^ and 324^th^ position had the minimum score (-2.489) and the maximum score (2.422) (**Figure 2B**). Moreover, more scores fell below zero, indicating that the protein is mainly hydrophilic.

Based on the prediction of SOPMA, random coil in the secondary structure of PtCHS protein accounted for 33.25%, α-helix 44.25%, extended strand 15.6%, and β-turn 6.91%, along with irregular coils and extended chains scattered in the whole protein structure (**Figure 2A**). The α-helix was the main structural component of the secondary structure of PtCHS protein. The tertiary structure prediction of PtCHS protein further showed that the protein was dominated by α-helices and random coils (**Figure 2C**), which was consistent with our secondary structure prediction. Based on the analysis of WoLF PSORT, the sub-cellular localization of the protein was in chloroplast, cytoplasm, and nucleus.

### Phylogenetic analysis of PtCHS

**Figure 3:**
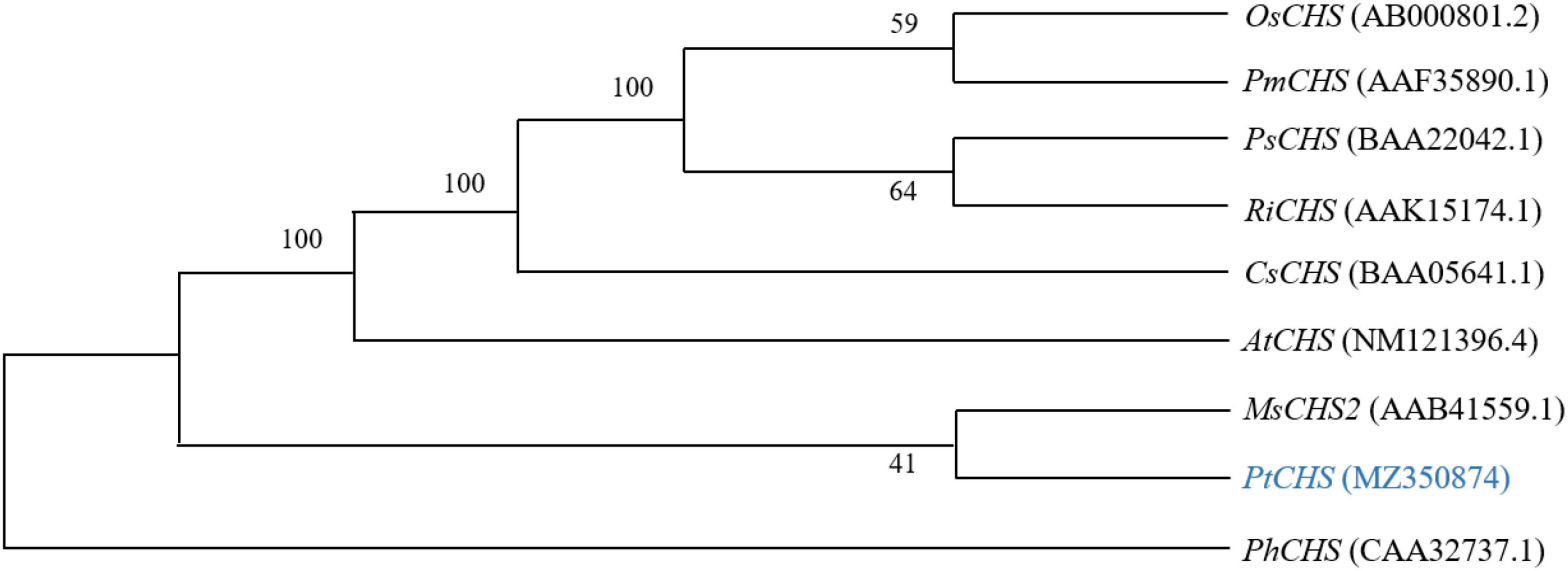
The phylogenetic tree analysis of PtCHS and CHS genes of other plants.

The alignment of the amino acid sequences of PtCHS and CHS of eight other plant species of NCBI showed that the sequence homology of *PtCHS* and *CHS* of other species was 92.49%, indicating that *PtCHS* gene has high sequence conservation. The phylogenetic tree showed that the closest homology of *PtCHS* was *MsCHS2* in *Medicago sativa* (**Figure 3**).

### Tissue-specific expression of *PtCHS* gene

**Figure 4:**
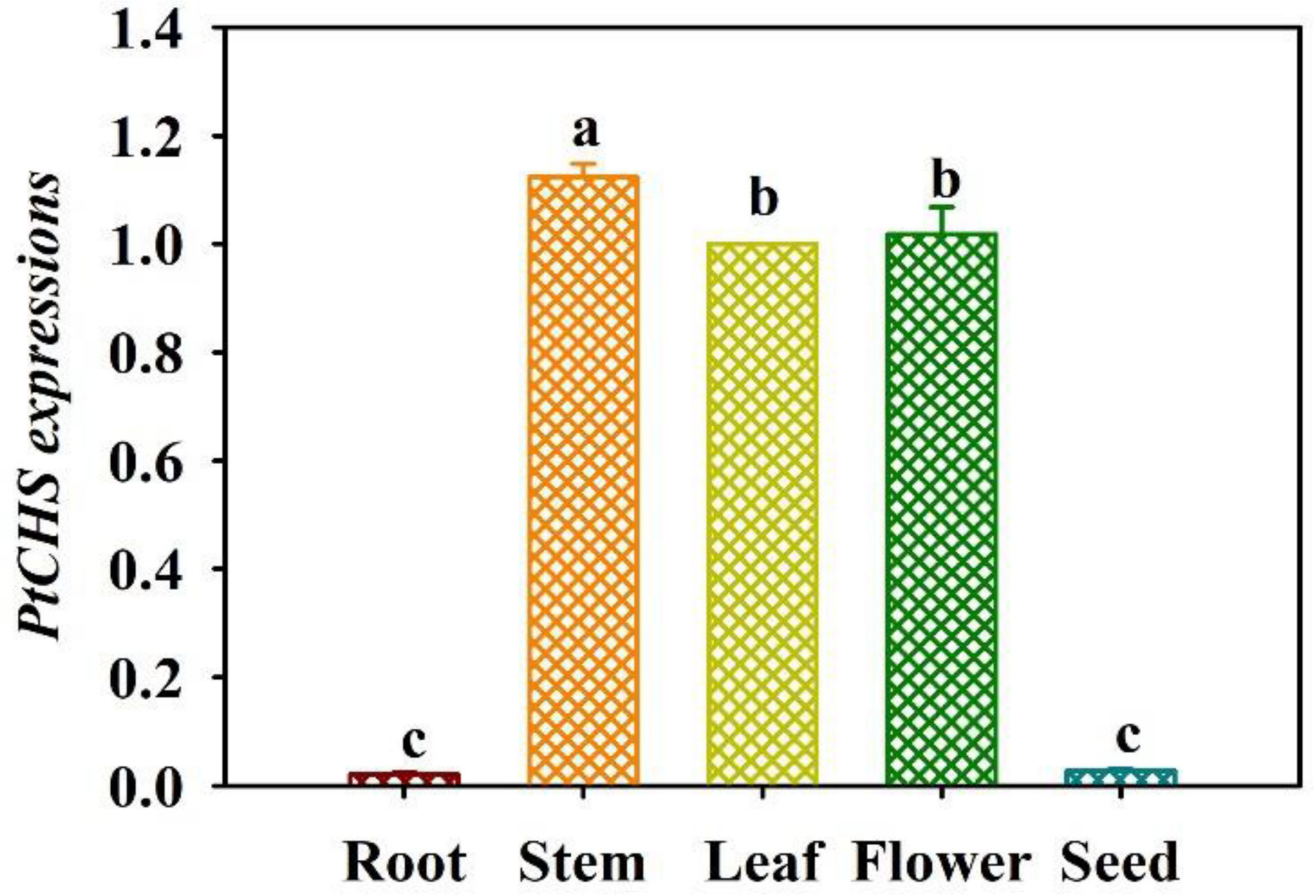
Tissue-specific expressions of *PtCHS* in trifoliate orange. Data (means ± SD, *n* = 4) followed by different letters above the bars indicate significant (*P* < 0.05) differences.

The qRT-PCR results showed that the expression of *PtCHS* gene in trifoliate orange was tissue-specific (**Figure 4**). *PtCHS* was expressed in the leaf, flower, stem, root, and seed. The highest expression of the gene was found in the stem, and the lowest in the root and seed, where the gene expression level of leaf was 53.2-fold higher than that of root.

### Root mycorrhizal colonization and total biomass in response to soil water deficit and AMF inoculation

**Figure 5:**
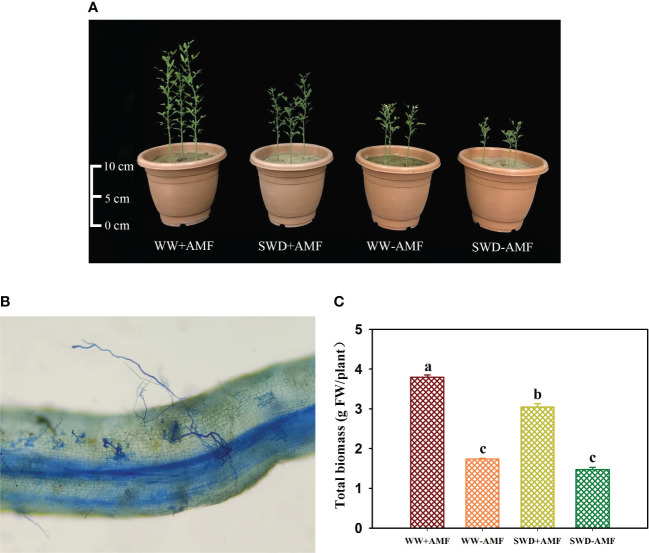
Changes in plant growth performance **(A)**, root mycorrhizal colonization **(B)**, and total biomass **(C)** of trifoliate orange in response to soil water deficit and *Funneliformis mosseae* inoculation. Abbreviations: WW+AMF, plants inoculated with *F mosseae* under well-watered; WW-AMF, plants inoculated without *F mosseae* under well-watered; SWD+AMF, plants inoculated with *F mosseae* under soil water deficit; SWD-AMF, plants inoculated without *F mosseae* under soil water deficit. Data (means ± SD, *n* = 4) followed by different letters above the bars indicate significant (*P* < 0.05) differences between treatments.

*F*. *mosseae* could colonize the roots of trifoliate orange seedlings, and the root mycorrhizal colonization was 38.5%−58.7%, accompanied by higher mycorrhizal colonization appearing under WW versus SWD conditions (**Figure 5B**). In addition, SWD significantly inhibited the growth performance of trifoliate orange seedlings, while *F*. *mosseae* inoculation improved plant growth response (**Figure 5A**). SWD treatment significantly reduced total biomass production of non-mycorrhizal and mycorrhizal plants by 18.58% and 24.48%, respectively, compared with WW treatment (**Figure 5C**). Nevertheless, the total biomass was increased by *F*. *mosseae* inoculation by 117.98% under WW and 107.64% under SWD, respectively.

### Relative expressions of *PtCHS* in response to soil water deficit and AMF inoculation

**Figure 6:**
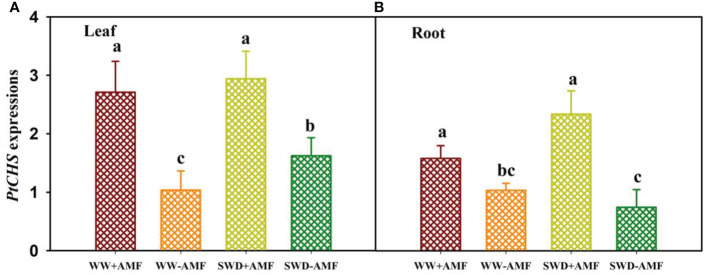
*PtCHS* gene expressions in leaf **(A)** and root **(B)** of trifoliate orange seedlings in response to soil water deficit and *Funneliformis mosseae* inoculation. Data (means ± SD, *n* = 4) followed by different letters above the bars indicate significant (*P* < 0.05) differences between treatments. See **Figure 5** for the abbreviations.

SWD and AMF (*F*. *mosseae*) inoculation affected relative expressions of *PtCHS* in leaves and roots (**Figures 6A, B**). Compared with the WW treatment, the SWD only up-regulated expressions of *PtCHS* in leaves of non-AMF-inoculated plants (**Figure 6A**). Compared with non-AMF inoculation, AMF inoculation up-regulated expressions of *PtCHS* under both WW and SWD: 1.62- and 0.81-fold higher in leaf and 0.53- and 2.14-fold higher in root, respectively (**Figures 6A, B**).

### CHS activity in response to soil water deficit and AMF inoculation

**Figure 7:**
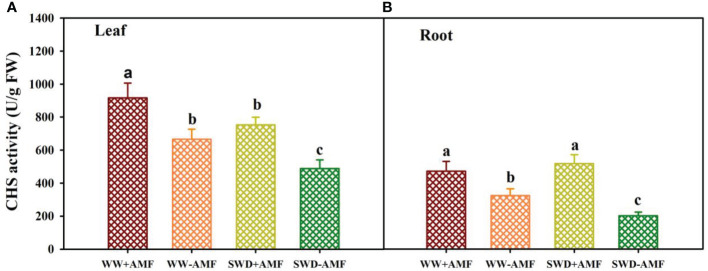
CHS activity in leaf **(A)** and root **(B)** of trifoliate orange seedlings in response to soil water deficit and *Funneliformis mosseae* inoculation. Data (means ± SD, *n* = 4) followed by different letters above the bars indicate significant (*P* < 0.05) differences between treatments. See **Figure 5** for the abbreviations.

SWD and AMF inoculation significantly altered CHS activity in leaves and roots of trifoliate orange seedlings (**Figures 7A, B**). SWD reduced CHS activity in leaves of mycorrhizal plants by 17.76%, along with no significant effect on roots. Nevertheless, SWD reduced CHS activity in leaves and roots of non-mycorrhizal plants by 26.67% and 37.60%, respectively, compared with WW treatment. Compared with non-inoculated treatment, AMF inoculation significantly increased the CHS activity of leaves and roots by 37.69% and 45.75% under WW conditions and by 54.42% and 156.47% under SWD conditions, respectively.

### Total flavonoid content and its correlation with PtCHS expressions

**Figure 8:**
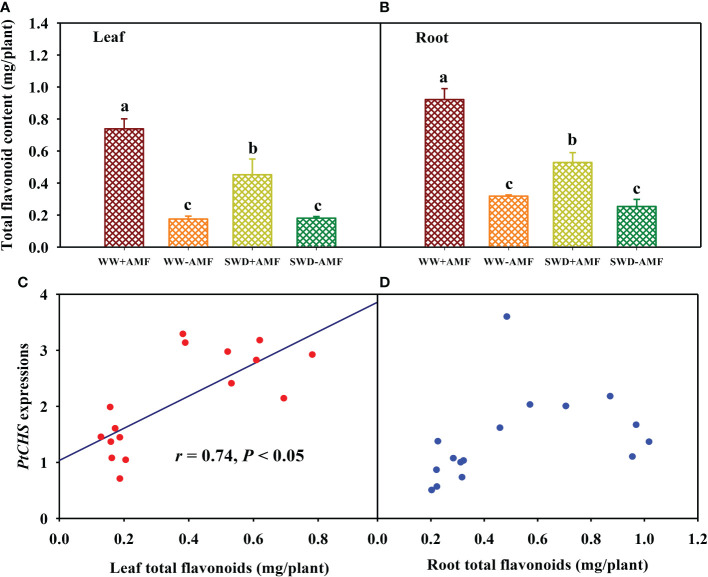
Changes in total flavonoid content in leaf **(A)** and root **(B)** of trifoliate orange in response to soil water deficit and *Funneliformis mosseae* inoculation and their correlation with *PtCHS* expressions in leaf **(C)** and root **(D)**. Data (means ± SD, *n* = 4) followed by different letters above the bars indicate significant (*P* < 0.05) differences between treatments. See **Figure 5** for the abbreviations.

SWD did not significantly affect total flavonoid content in leaves and roots of plants inoculated without *F*. *mosseae*, but significantly reduced total flavonoid content in leaves and roots of *F*. *mosseae*-inoculated plants by 0.39- and 0.43-fold, respectively, compared with WW treatment (**Figures 8A, B**). On the other hand, inoculation with *F*. *mosseae* also significantly increased total flavonoid content of plants under both WW and SWD conditions, where it increased by 3.20- and 1.50-fold in leaves and 1.90- and 1.09-fold in roots, respectively. Correlation analysis showed that total flavonoid content was a significantly positive correlation with *PtCHS* expression in leaves (**Figure 8C**), along with no significant correlation in roots (**Figure 8D**).

## Discussion

Hahlbrock and Kreuzaler (1972) firstly extracted the CHS from suspension cells of parsley. Subsequently, many scholars have carried out studies on cloning CHS gene of various plants (Liu et al., 2011; Vadivel et al., 2018). In this study, a *PtCHS* gene was cloned from trifoliate orange, with a full length of 1156 bp, encoding 391 amino acids. This gene was highly homologous to *CHS* of other plants with 92.49%, showing a high degree of conservative property and further indicating that this gene is stable, consistent with earlier results (Pang et al., 2005; Wang et al., 2017).

Earlier studies showed that most of the *CHS* genes were located in the cytoplasm at the sub-cellular level (Wang et al., 2017; Vadivel et al., 2018). This study also predicted that PtCHS protein may be located in chloroplasts, cytoplasm, and nucleus, indicating that CHS protein widely presents in cell organelles. In addition, *PtCHS* gene expression was highest in stem, followed by flower and leaf, and very low in root and seed, indicating that *PtCHS* expression is tissue-specific. Pang et al. (2005) also found *CHS* expression in stem and leaf of ginkgo, and no expression was detected in roots. In addition, *CHS* expressions in plants vary in developmental periods: in early stages of plants, *CHS* expressions appear in leaves, whereas in mature plants *CHS* gene is mainly expressed in flowers, indicating that *CHS* expression in plants is mainly in aboveground parts (Knogge et al., 1986).

It has been shown that expression of *CHS* genes could be induced by external environments (Koes et al., 1994; Cushnie and Lamb, 2011; Singh and Kumaria, 2020). In the present study, SWD treatment increased the expression of *PtCHS* in leaves of non-mycorrhizal plants. Ahmed et al. (2021) also reported the up-regulated expression of *CHS* in leaves of poplar after SWD, and the up-regulated magnitude of the gene was increased with the extension of SWD. In addition, our study also indicated that *F*. *mosseae* inoculation increased expressions of *PtCHS* and CHS activity in leaves and roots regardless of soil water regimes. Moreover, the up-regulated magnitude of *PtCHS* by mycorrhization was higher under WW than under SWD, which may be due to the inhibition of root fungal colonization in SWD (Ding et al., 2022), thus reducing the efficiency of mycorrhizal fungi. Meanwhile, *PtCHS* expressions were significantly positively correlated with leaf total flavonoid content. Wang et al. (2010) also found a significantly positive correlation between *CHS* and total flavonoid concentration in fruits of Guoqing No. 4 satsuma mandarin. In tomato, Aseel et al. (2019) reported the increase in total flavonoids in leaves after AMF inoculation and/or infection with *Tomato Mosaic Virus*. They also found that inoculation with AMF decreased the expression of *CHS*, but *Tomato Mosaic Virus* infection did not change *CHS* expression, along with induced expression of *CHS* after double inoculation of AMF and *Tomato Mosaic Virus*.

The present study also observed that SWD did not alter leaf and root total flavonoid content of non-AMF-treated plants, while it significantly reduced leaf and root total flavonoid content of AMF-treated plants. Earlier studies on alfalfa also showed that total flavonoids increased first and then decreased with the increase of PEG concentration (Li et al., 2020). In the Chuanqiao 1 variety of Tartary buckwheat, total flavonoids in leaves and grains were not changed after 7 days of drought stress, but leaf total flavonoids were decreased after 14 days of drought (Ouyang et al., 2020). This indicated that the variation of total flavonoids under SWD was affected by stress intensity, stress time, plant variety, and AMF. It is necessary to further use the targeted metabolome to determine which flavonoids in the total flavonoids can be responded to SWD in trifoliate orange. In addition, total flavonoid content was reduced in AMF-inoculated plants under SWD versus WW conditions, which may be because plants consume certain flavonoids to maintain mycorrhizal activity under SWD (Tian et al., 2021). A significantly positive correlation (*r* = 0.95, *P* < 0.01) was found between root mycorrhizal colonization and root total flavonoid content, suggesting the important role of flavonoids in mycorrhizae.

In our study, mycorrhiza-inoculated plants recorded dramatically higher total flavonoid content than mycorrhiza-uninoculated plants under both WW and SWD conditions. Similar result was observed in *Pistacia vera* inoculated with *Glomus etunicatum* and *Nicotiana tabacum* colonized by *G*. *versiforme* under SWD (Abbaspour et al., 2012; Begum et al., 2019). Higher total flavonoids in mycorrhizal versus non-mycorrhizal plants suggested that mycorrhizal plants under SWD have greater capacity to remove reactive oxygen species than non-mycorrhizal plants (Liu et al., 2022). Nevertheless, Amiri et al. (2017) reported a significant increase in total flavonoid levels in leaves of *Pelargonium graveolens* by *F*. *mosseae* under WW, but not SWD. On the other hand, the present study also showed a significantly positive correlation between total flavonoid content and *PtCHS* expression only in leaves, but not in roots, suggesting that there may be different mechanisms for mycorrhiza-induced changes in total flavonoids between leaves and roots. In addition, the up-regulated magnitude of root *PtCHS* expression levels triggered by AMF inoculation was higher under SWD conditions than under WW conditions, while the elevated magnitude of total flavonoid content in roots caused by AMF inoculation was higher under WW conditions than under SWD conditions. Therefore, *CHS* was not the most critical factor for mycorrhizal enhancement of total flavonoids under SWD conditions. Of course, the production of flavonoids depends on a number of enzymes in biological pathways of flavonoids, such as phenylalanine ammonia-lyase, 4-coumarate CoA ligase, and chalcone synthase, besides CHS (Ma et al., 2014). In wheat, *CHS* and *CHI* can be jointly regulated in response to SWD (Ma et al., 2014). Therefore, responsive patterns of more flavonoid biosynthesis genes need to be analyzed under SWD and mycorrhization conditions. It is concluded that AMF up-regulates the expression of *CHS* in the host, especially under abiotic and biotic stress conditions, thus showing the important characteristics of mycorrhizal tolerance to stress.

## Conclusions

In this study, a *CHS* gene, named *PtCHS*, was cloned from the genome-wide of trifoliate orange, with 92.49% homology with other species. This gene had tissue-specific expression, along with high expression in aboveground parts such as leaf, flower, and stems. *PtCHS* was regulated by SWD and AMF inoculation, where *F*. *mosseae* up-regulated *PtCHS* expressions in leaves and roots, independent on soil water status, providing the support for total flavonoid production in plants, especially leaves. However, more work is needed around which flavonoid components are modulated by mycorrhizal fungi and which flavonoid synthesis genes are affected by SWD and mycorrhization.

## Data availability statement

The original contributions presented in the study are included in the article/supplementary material. Further inquiries can be directed to the corresponding author.

## Author contributions

Conceptualization, Q-SW and Y-NZ; data curation, ZL, SC, and X-QL; methodology, SC; resources, Q-SW; supervision, Q-SW and Y-NZ; writing—original draft, ZL; writing—review and editing, AH, A-BA-A, KK, KA, EA, Y-NZ, and Q-SW. All authors have read and agreed to the published version of the manuscript.
